# Comparison Between Bicuspid and Tricuspid Aortic Regurgitation: Presentation, Survival, and Aorta Complications

**DOI:** 10.1016/j.jacasi.2022.02.012

**Published:** 2022-04-02

**Authors:** Li-Tan Yang, Hao-Yun Lo, Chien-Chang Lee, Masaaki Takeuchi, Tzu-Chun Hsu, Chieh-Mei Tsai, Hector I. Michelena, Maurice Enriquez-Sarano, Yih-Sharng Chen, Wen-Jone Chen, Yi-Lwun Ho

**Affiliations:** a Division of Cardiology, Department of Internal Medicine, National Taiwan University Hospital, Taipei, Taiwan; b Cardiovascular Center, National Taiwan University Hospital, Taipei, Taiwan; c Department of Internal Medicine, College of Medicine, National Taiwan University, Taipei, Taiwan; d Telehealth Center, National Taiwan University Hospital, Taipei, Taiwan; e Department of Emergency Medicine, National Taiwan University Hospital, Taipei, Taiwan; f Center of Intelligent Healthcare, National Taiwan University Hospital, Taipei, Taiwan; g Department of Laboratory and Transfusion Medicine, Hospital of University of Occupational and Environmental Health, School of Medicine, Kitakyushu, Japan; h Department of Cardiovascular Disease, Mayo Clinic, Rochester, Minnesota, USA; i Department of Surgery, National Taiwan University Hospital, Taipei, Taiwan

**Keywords:** aortic regurgitation, bicuspid aortic valve, survival, tricuspid aortic valve, AR, aortic regurgitation, AVS, aortic valve surgery, BAV, bicuspid aortic valve, BP, blood pressure, BSA, body surface area, CCI, Charlson Comorbidity Index, LAVi, left atrial volume index, LV, left ventricular, LVEDD, left ventricular end-diastolic dimension, LVEF, left ventricular ejection fraction, LVESD, left ventricular end-systolic dimension, LVESDi, left ventricular end-systolic dimension index, RL, right–left coronary, TAV, tricuspid aortic valve, TTE, transthoracic echocardiogram, SOV, sinus of Valsalva

## Abstract

**Background:**

Although the Asian population is growing globally, data in Asian subjects regarding differences between bicuspid aortic valve (BAV) and tricuspid aortic valve (TAV) in aortic regurgitation (AR) remain unexplored.

**Objectives:**

The aim of this study was to examine differences between Asian BAV-AR and TAV-AR in significant AR, including aorta complications.

**Methods:**

The study included 711 consecutive patients with chronic moderate to severe and severe AR from 2008 to 2020. Outcomes included all-cause death, aortic valve surgery (AVS), and incidence of aortic dissection (AD).

**Results:**

There were 149 BAV-AR (mean age: 48 ± 16 years) and 562 TAV-AR (mean age: 68 ± 15 years; *P <* 0.0001) patients; baseline indexed left ventricle and indexed aorta size were larger in TAV-AR. Total follow-up was 4.8 years (IQR: 2.0-8.4 years), 252 underwent AVS, and 185 died during follow-up; 18 cases (only 1 BAV) of AD occurred, with a mean maximal aorta size of 60 ± 9 mm. The 10-year AVS incidence was higher in TAV-AR (51% ± 4%) vs BAV-AR (40% ± 5%) even after adjustment for covariates (*P <* 0.0001). The 10-year survival was higher in BAV-AR (86% ± 4%) vs TAV-AR (57% ± 3%; *P <* 0.0001) and became insignificant after age adjustment *(P =* 0.33). Post-AVS 10-year survival was 93% ± 5% in BAV-AR and 78% ± 5% in TAV-AR, respectively *(P =* 0.08). The 10-year incidence of AD was higher in TAV-AR (4.8% ± 1.5%) than in BAV-AR (0.9% ± 0.9%) and was determined by aorta size ≥45 mm (*P* ≤ 0.015). Compared with an age- and sex-matched population in Taiwan, TAV-AR (HR: 3.1) had reduced survival (*P <* 0.0001).

**Conclusions:**

Our findings suggest that TAV-AR patients were at a later stage of AR course and had a high AD rate as opposed to BAV-AR patients in Taiwan, emphasizing the importance of early referral for timely management. Surgery on the aorta with a lower threshold in TAV-AR should be considered.

Bicuspid aortic valve (BAV) is the most frequently seen congenital heart defect^1^ and represents an increasing etiology of hemodynamically significant aortic regurgitation (AR), which is the third most common valvular heart disease.^2^ Compared with patients with tricuspid aortic valve (TAV), patients with BAV-AR are distinctly different: they are more than a decade younger,^2^ have more mixed mechanisms of AR (including cusp prolapse and root dilatation), have larger aortic annulus, and exhibit better survival.^3^ Despite these inherent differences, publications comparing BAV vs TAV in hemodynamically significant AR are scarce,^3^ and Asian data are especially lacking.^4^ Interethnic differences in BAV have been reported,^5^ suggesting that findings from Western populations may not hold true completely in those of Asian ancestry.

The Asian population is growing and accounts for 60% of the global population. However, data that serve as the backbone of practice guidelines, which most Asian clinicians abide by, frequently come from Western populations. Understanding contemporary profiles of valvular heart disease in Asia helps to reduce global disease burden and promote health; hence, there is an urgent need to report Asian data. In Taiwan, health care is provided by National Health Insurance^6^; because of its relatively low cost and easy access regardless of socioeconomic background, transthoracic echocardiogram (TTE) examination for valvular heart disease is commonly arranged and affordable.

The current study compares consecutive patients with TAV-AR and BAV-AR identified from the TTE database in a tertiary university hospital in Taiwan regarding presentation, determinants for symptoms, surgical indication, surgical incidence, survival, and feared aorta complications.

## Methods

### Study population and clinical data

Between 2008 and 2020, all consecutive patients aged ≥18 years with chronic moderate to severe and severe AR according to TTE were retrospectively identified from an electronic echo database. All cases were manually reviewed to determine eligibility. Exclusion criteria included: mitral stenosis/regurgitation and aortic stenosis more than mild, prior mitral/aortic surgery, complex cyanotic congenital heart disease, and acute AR (dissection, trauma, and active endocarditis) (Supplemental Figure 1). After exclusions, 711 patients constituted the study cohort. All patients had comprehensive cardiology and/or cardiovascular surgery evaluations within 30 days of TTE. Baseline New York Heart Association functional class data, independently recorded by treating physicians prospectively, were meticulously abstracted from the electronic medical record or paper charts. Comorbid conditions recorded during AR consultation were manually extracted. A patient was considered to have Marfan syndrome if confirmed by genetic testing or if manifesting classic clinical stigmata of the disease, as judged by the treating physician. Charlson Comorbidity Index (CCI) was computed. Our institutional review board approved this study.

### Echocardiography and mechanisms of AR

In patients with multiple TTEs, the first eligible study was used for analysis. TTE was performed by trained sonographers using commercially available echo systems and de novo reviewed by cardiologists with level III echocardiography training (L.-T.Y.). Left ventricular (LV) volumes were derived from a biplane disk summation method or single plane if biplane not feasible.^7^ Other chamber quantification and semi-quantitative measurements for AR (vena contracta width, time-velocity integral of descending aorta reversed-flow) were performed by using an integrated, comprehensive approach according to guidelines.^7^^,^^8^ In patients with BAV, cusp fusion (right–left coronary [RL], right noncoronary, and left noncoronary) was recorded.^2^ A de novo review of TTE was conducted to define mechanisms of AR (single or mixed), including cusp prolapse, cusp restriction/retraction, and dilatation of aortic root as described previously,^2^ as well as by review of all available surgical reports, pathology reports, and medical records.

### Primary endpoints

We compared BAV-AR vs TAV-AR regarding all-cause mortality during total follow-up (observation stopped at death or last follow-up), under medical surveillance (observation stopped at aortic valve surgery [AVS], death, or last follow-up) and post-AVS. Overall survival was also compared with expected survival from an age-and sex-matched general population in Taiwan. Mortality status, dates of death, and cause of death were retrieved from medical records and Taiwan’s National Health Insurance Research Database.

### Secondary endpoints

We compared BAV-AR vs TAV-AR regarding: 1) surgical indications (symptoms, LV ejection fraction [LVEF] <50%, aorta surgery, LV end-systolic dimension [LVESD] >50 mm, LVESD index [LVESDi] >25 mm/m^2^, and LV end-diastolic dimension[LVEDD] >65 mm) based on guidelines^9^; 2) cumulative incidence of AVS (ie, aortic valve repair or replacement); 3) LV recovery defined as LV reverse remodeling between presurgical and post-AVS TTE (within 6-18 months’ post-AVS); and 4) incidence of aortic dissection (AD). Information regarding AVS status (indications, valve used, and concomitant surgical procedures) was ascertained from medical records and telephone interview, if necessary.

### Statistical analysis

Continuous variables, expressed as mean ± SD or median (IQR) according to data distribution, were compared by using the Student’s *t-*test or Wilcoxon rank sum test whenever appropriate. Categorical data, presented as percentages, were compared by using chi-square tests. Linear and logistic regression models were used to compare continuous and categorical variables, respectively. Kaplan-Meier curves were used to estimate survival over time, including the survival from our cohort and the expected survival in which differences in survival were tested by using the log-rank test. Expected survival was derived based on the survival of the control group (10 times the number of BAV or TAV subgroup) in the general population matched by age and sex.^10^ The endpoints of mortality and surgical incidence between BAV and TAV were analyzed by using the Cox proportional hazards model, while adjusting for age, sex, CCI, LVEF, and New York Heart Association functional class in multivariable analysis (additionally adjusted for time-dependent AVS for “survival during total follow-up”).

All statistical analyses were performed by using commercially available software (JMP 16, SAS Institute Inc; R version 4.0.5, The R foundation for Statistical Computing). A 2-sided *P* value of <0.05 was considered statistically significant.

## Results

### Baseline characteristics

Baseline characteristics of this cohort (n = 711), including 149 (21%) BAV-AR and 562 (79%) TAV-AR patients, are shown in Table 1. At baseline, those with BAV were 2 decades younger, mostly male, had larger body surface area (BSA), fewer comorbidities, smaller left atrial volume index (LAVi), and lower maximal pressure-gradient of tricuspid regurgitation. After BSA normalization, patients with TAV had larger indexed dimensions of the left ventricle, sinus of Valsalva (SOV), and ascending aorta, suggesting that TAV-AR could have more advanced LV remodeling. Notably, linear regression analysis revealed that BAV-AR had higher trans-AV velocity (β estimate adjusted for age, sex, stroke volume, and LVEF: 0.26; *P <* 0.0001), possibly reflecting its intrinsic stenosis.^11^ The BAV-fusion type was discernable in 148 patients, including RL cusp fusion in 107, right-noncoronary cusp fusion in 34, and left-noncoronary cusp fusion in 7 patients. RL cusp fusion had similar dimensions of SOV and ascending aorta (both; *P ≥* 0.32) but larger aortic annulus *(P =* 0.017) compared with non–RL cusp fusion BAV-AR.

**Table 1:** Baseline Characteristics and Echocardiographic Parameters in All Patients

	Total (N = 711)	BAV (n = 149)	TAV (n = 562)	*P* Value
Age, y	63 ± 17	48 ± 16	68 ± 15	**<0.0001**
Female	158 (23)	14 (9)	148 (26)	**<0.0001**
Body surface area, m^2^	1.70 ± 0.20	1.78 ± 0.19	1.68 ± 0.20	**<0.0001**
Systolic blood pressure, mm Hg	135 ± 18	136 ± 18	135 ± 18	0.49
Diastolic blood pressure, mm Hg	66 ± 12	67 ± 12	65 ± 12	0.05
Pulse pressure, mm Hg	69 ± 20	68 ± 18	69 ± 20	0.55
Hypertension	431 (61)	74 (51)	357 (64)	**0.005**
Hyperlipidemia	126 (18)	25 (18)	101 (18)	0.89
Diabetes mellitus	55 (18)	8 (6)	47 (8)	0.25
Atrial fibrillation	51 (7)	3 (2)	48 (9)	**0.001**
Connective tissue disease	46 (6)	4 (3)	42 (8)	**0.027**
Marfan syndrome	26 (4)	3 (2)	23 (4)	0.19
Coronary artery disease	171 (24)	18 (13)	153 (27)	**<0.0001**
Remote history of infective endocarditis	22 (3)	7 (5)	15 (3)	0.17
Charlson Comorbidity Index	1.40 ± 1.77	0.82 ± 1.41	1.55 ± 1.82	**<0.0001**
Aortic valve surgery	252 (35)	51 (34)	201 (36)	0.72
New York Heart Association functional class				**<0.0001**
I	370 (52)	100 (67)	270 (48)	
II	253 (36)	40 (27)	213 (38)	
III + IV	76 (11)	4 (3)	72 (13)	
Undetermined	12 (1)	5 (3)	7 (1)	
Echo parameters				
BAV fusion type^a^				
RL (n = 107)	─	72%	─	─
RN (n = 34)	─	23%	─	─
LN (n = 7)	─	5%	─	─
LV ejection fraction, %	63 ± 10	65 ± 9	62 ± 10	**0.001**
LV end-diastolic dimension, mm	60 ± 7	61 ± 7	60 ± 7	0.05
LV end-diastolic dimension index, mm/m^2^	35.4 ± 4.7	34.4 ± 4.4	35.7 ± 4.8	**0.002**
LV end-systolic dimension, mm	39 ± 8	39 ± 7	39 ± 8	0.69
LV end-systolic dimension index, mm/m^2^	23.0 ± 4.9	21.9 ± 4.3	23.3 ± 5.0	**0.0004**
LV end-systolic dimension index >25 mm/m^2^	180 (25)	26 (17)	154 (27)	**0.010**
LV end-diastolic volume index, mL/m^2^ (n = 700)	100 ± 37	107 ± 36	98 ± 37	**0.007**
LV end-systolic volume index, mL/m^2^ (n = 698)	44 ± 24	46 ± 21	43 ± 25	0.17
Peak trans-aortic valve velocity, m/s	1.9 ± 0.5	2.1 ± 0.6	1.8 ± 0.5	**<0.0001**
LAVi, mL/m^2^ (n = 668)	30 ± 14	27± 13	31 ± 14	**0.001**
TR PG, mm Hg (n = 661)	25 ± 8	23± 6	26 ± 9	**<0.0001**
E/e′ (n = 456)	14 ± 6	13± 5	14 ± 6	0.18
AR vena contracta, mm (n = 530)	7.0 ± 1.9	6.9± 1.6	7.0 ± 1.9	0.73
AR pressure-half time, ms (n = 289)	342 (277-414)	309 (272-367)	345 (274-419)	0.32
Dimensions of aorta				
Annulus, mm (n = 687)	23.5 ± 3.4	25.9 ± 3.7	22.9 ± 3.0	**<0.0001**
Indexed annulus (n = 687)	13.9 ± 1.9	14.7 ± 2.3	13.7 ± 1.9	**<0.0001**
Sinus of Valsalva, mm(n = 681)	41.7 ± 8.4	40.3 ± 5.6	42.0 ± 8.9	**0.003**
Indexed sinus of Valsalva (n = 681)	24.6 ± 5.0	22.8 ± 3.7	25.1 ± 5.2	**<0.0001**
Sinus of Valsalva ≥45 mm (n = 681)	176 (26)	22 (16)	154 (28)	**0.001**
Mid-ascending aorta, mm (n = 349)	44.0 ± 8.1	42.6 ± 7.7	44.3 ± 8.1	0.13
Indexed mid-ascending aorta (n = 349)	26.1 ± 5.5	23.9 ± 4.8	26.5 ± 5.6	**0.0003**
Mid-ascending aorta ≥45 mm (n=349)	147 (42)	21 (37)	126 (43)	0.37

Values are mean ± SD, n (%), or median (IQR), unless otherwise indicated.

AR = aortic regurgitation; BAV = bicuspid aortic valve; E/e′ = peak mitral inflow velocity to early diastolic mitral annular velocity ratio; LAVi = left atrial volume index; LV = left ventricular; TAV = tricuspid aortic valve; TRPG = tricuspid regurgitation maximal pressure gradient.

a The average age of those with right–left coronary (RL) cusp fusion, right noncoronary (RN) cusp fusion, and left noncoronary (LN) cusp fusion were: 49 ± 16, 47 ± 16, 53 ± 26 years, respectively (ANOVA; *P =* 0.73). **Bold** value indicates *P* ≤ 0.05.

### Mechanisms of AR

Analysis was performed in 667 patients whose AR mechanisms were recognizable via TTE. Compared with patients with TAV-AR, BAV-AR patients had a higher prevalence of mixed mechanisms (60% vs 26%) and any cusp prolapse (57% vs 12%) (both: *P ≤* 0.0001) but similar chances of cusp restriction/retraction (18% vs 22%; *P =* 0.30) and any dilatation of annulus or aortic root (84% vs 88%; *P =* 0.24).^2^

### Baseline symptomatic status and echocardiographic findings

The relationships between baseline symptoms and chamber remodeling as well as AR mechanisms are shown in Supplemental Table 1**.** Symptomatic BAV-AR patients were older, had higher systolic blood pressure (BP) and pulse pressure, lower LVEF, and more cusp restriction with a trend toward larger LAVi and maximal tricuspid regurgitation pressure gradient. Multivariate determinants for symptomatic BAV-AR were: older age (odds ratio: 1.04; 95% CI: 1.01-1.08; *P =* 0.0004), higher systolic BP (odds ratio: 1.03; 95% CI: 1.00-1.05; *P =* 0.006), and lower LVEF (odds ratio per 10% increase: 0.59; 95% CI: 0.35-1.00; *P =* 0.03). In symptomatic TAV-AR, in addition to larger LAVi, higher E/e′, and tricuspid regurgitation pressure gradient, LV dimensions/volumes were proportionally related to symptoms. Multivariate predictors of symptomatic TAV-AR were older age, higher pulse pressure, larger LAVi, and one of the following: low LVEF, larger LVESDi, or larger LVEDDi (all: *P ≤* 0.03).

### Surgical incidence, indications, and lv reverse remodeling

**Figure 1:**
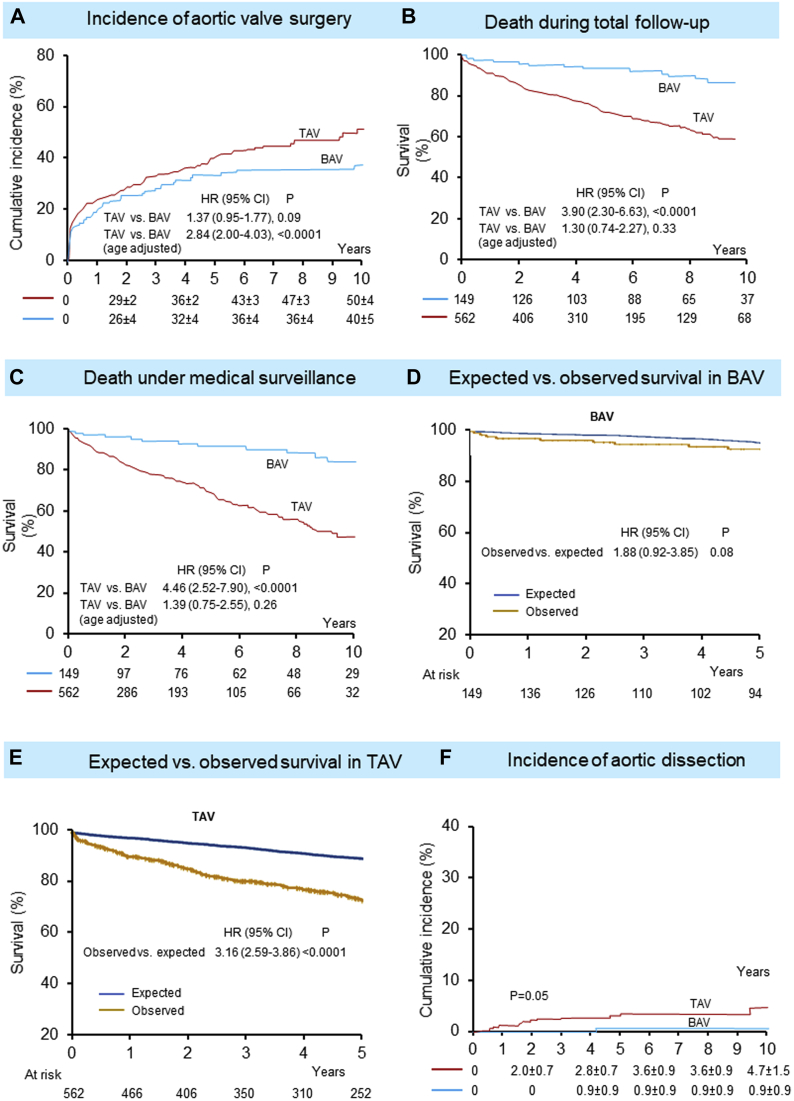
Survival Differences Between BAV-AR and TAV-AR Tricuspid aortic valve in aortic regurgitation (AR) (TAV-AR) patients had higher age-adjusted incidence of aortic valve surgery **(A)**. Bicuspid aortic valve in AR (BAV-AR) patients had better survival during total follow-up **(B)** and under medical surveillance **(C)**; these survival differences attenuated after adjustment for age. Compared with an age- and sex-matched general population, BAV-AR patients **(D)** had similar survival, although TAV-AR patients **(E)** had decreased survival during total follow-up. **(F)** The cumulative incidence of aortic dissection was higher with TAV-AR.

In total, 252 (35%) patients underwent AVS, including 51 BAV patients and 201 TAV patients; no patients had received transcatheter aortic valve replacement. The 10-year AVS incidence in TAV vs BAV was 51% ± 4% vs 40% ± 5% *(P =* 0.09), respectively; TAV patients had 2.84-fold risk of having AVS after adjustment for age (Figure 1A) and after additional adjustment for sex, CCI, LVEF, and New York Heart Association functional class (HR: 2.85; 95% CI: 1.98-4.10; *P <* 0.0001). Median time from baseline TTE to AVS was similar between BAV and TAV (9.6 [IQR: 1-32] months vs 4.3 [IQR: 0.7-26] months; *P =* 0.43). As for BAV phenotypes, RL cusp fusion was associated with a trend toward higher AVS incidence (HR: 1.76; 95% CI: 0.87-3.52; *P =* 0.09). For surgical patients with concomitant aorta surgery (46%), BAV-AR patients had larger annulus and annulus/BSA, whereas TAV-AR patients had larger SOV and SOV/BSA (Table 2). Comparison between those with and without concomitant aorta surgery is shown in Supplemental Table 2; the former had a higher prevalence of connective tissue disease and Marfan syndrome, were less symptomatic, and apparently had larger aorta size.

**Table 2:** Surgical Indications According to Guidelines and Types of Surgery (N = 252)

	BAV (n = 51)	TAV (n = 201)	*P* Value
Surgical indications, n = 252			**0.0002**
Symptoms	27 (53)	166 (83)	
LV ejection fraction <50%	1 (2)	1 (0.5)	
Aortic aneurysms	4 (8)	13 (6.5)	
LV end-systolic dimension (index) >50 mm (25 mm/m^2^)	7 (13)	13 (6.5)	
LV end-diastolic dimension >65 mm	6 (12)	3 (1.5)	
Early surgery without aforementioned indications	6 (12)	5 (2.5)	

	**BAV (n = 51)**	**TAV (n = 198)**	
Surgical procedures,^a^ n = 249			
Aortic valve repair	1 (–)	7 (–)	─
Concomitant aorta surgery	14 (27)	100 (50)	**0.003**
Concomitant coronary artery bypass grafting	4 (8)	25 (12)	0.33
Mechanical valve, n = 241	21 (43)	57 (30)	0.08
Valve size^b^			0.25
<23 mm	2 (5)	18 (11)	
23-24 mm	15 (36)	76 (44)	
25 mm	24 (57)	71 (41)	
>25 mm	1 (2)	7 (4)	
Aorta size at surgery for aorta			
Annulus, mm	28.5 ± 3.50	24.6 ± 3.65	**0.001**
Indexed annulus, mm/m^2^	15.2 ± 1.8	13.9 ± 2.1	**0.03**
Sinus of Valsalva, mm	46.5 ± 7.3	53.6 ± 11.8	**0.006**
Indexed sinus of Valsalva, mm/m^2^	24.9 ± 4.3	30.4 ± 6.4	**0.0007**
Mid-ascending aorta, mm	51.5 ± 5.8	51.2 ± 10.9	0.87
Indexed mid-ascending aorta, mm/m^2^	28.2 ± 4.3	29.6 ± 7.8	0.37

Values are n (%) or mean ± SD. The study used 2014 American College of Cardiology/American Heart Association guidelines.

Abbreviations as in Table 1.

a Analysis excluded 3 patients: 1 underwent cardiac transplantation, and 2 died intraoperatively.

b Of 241 patients having aortic valve replacement, valve size were unknown in 27 patients who received aortic valve surgery other than at the study hospital. **Bold** value indicates *P* value ≤0.05.

Regarding surgical indications (Table 2), patients with BAV-AR were less operated on for symptoms but more so for LVESDi >50 mm (25 mm/m^2^), LVEDD >65 mm, and early surgery, compared with TAV-AR patients. The prevalence of LVESDi >25 mm/m^2^ was similar between BAV (16%) and TAV (17%) asymptomatic patients *(P =* 0.80). Concomitant aorta surgery was performed more in TAV-AR patients, reflected by their larger SOV. There were no differences regarding prosthetic valve size, although both were smaller than in patients from the United States (>40% used ≥27 mm prosthesis).^2^ BAV-AR had a trend toward more mechanical valve use *(P =* 0.08). All 30-day post-AVS mortality occurred in TAV-AR (n = 4).

Of 252 patients undergoing AVS, 133 (53%) had a follow-up TTE between 6 and 18 months post-AVS (median time: 12 [IQR: 9.5-14.5] months). Post-AVS LVEDD, LVESD, and LVESDi decreased significantly compared with pre-AVS TTE in both groups (all: *P <* 0.0001). Regarding LVEF, TAV-AR patients had a significantly improved post-AVS LVEF (59% ± 11% vs 65 ± 10%; *P <* 0.0001), and BAV-TR patients exhibited a trend toward improvement (62% ± 7% vs 64% ± 6%; *P =* 0.08) (Supplemental Figure 2).

### Overall survival, survival under medical surveillance, and post-avs survival

During a median follow-up of 4.8 years (IQR: 2.0-8.4 years), 185 patients died (28 died post-AVS), including 170 (30%) TAV and 15 (10%) BAV patients. The mortality follow-up was 100% by December 2020.

BAV-AR had significantly better 10-year survival both for the total follow-up (86% ± 4% vs 57% ± 3%; *P <* 0.0001) and follow-up under medical surveillance (85% ± 4% vs 47% ± 4%; *P <* 0.0001) as shown by Kaplan-Meier curves (Figures 1B to 1C), yet this survival difference disappeared after adjusting exclusively for age *(P =* 0.33 for total follow-up and *P =* 0.26 under medical surveillance) and after additional adjustment for sex, CCI, LVEF, New York Heart Association functional class, and time-dependent AVS (BAV vs TAV; *P =* 0.87 for total follow-up [Supplemental Table 3] and *P =* 0.83 under medical management). Adjustment for year of baseline TTE did not change the results. Also, Kaplan-Meier curves showed that those with AVS and aortic surgery had better overall survival, whereas those without AVS had the worst survival (Supplemental Figure 3A). Cox proportional hazards model revealed that baseline maximal aorta size (absolute or indexed to BSA) was independently associated with death under medical surveillance (Supplemental Table 4).

Compared with an age- and sex-matched general population in Taiwan, TAV-AR patients had 3.16-fold risk of death (95% CI: 2.59-3.86; *P <* 0.0001), whereas BAV-AR patients had a similar risk of death (HR: 1.88; 95% CI: 0.92-3.85; *P =* 0.08) at 5 years (Figures 1D-1E).

After AVS, 28 patients died at a median of 2.2 years (IQR: 0.3-5.0 years). BAV patients (93% ± 5%) had better 10-year post-AVS survival compared with TAV patients (78% ± 5%) *(P =* 0.08); however, after adjusting for age, survival was independent of valve anatomy (HR for BAV-AR: 0.74; 95% CI: 0.16-3.35; *P =* 0.69). Also, those with AVS and aortic surgery had better post-AVS survival compared with those with AVS alone, mostly driven by TAV-AR (Supplemental Figures 3B to 3C); after adjustment for age and CCI, AVS plus aorta surgery exhibited a trend toward better post-AVS survival (HR: 0.45; 95% CI: 0.19-1.08; *P =* 0.07) (Supplemental Table 4).

### Aortic dissection

During follow-up, AD occurred in 18 patients (type A in 17 patients and type B in 1 patient; average age: 63 ± 15 years; average BSA: 1.76 ± 0.24 m^2^; 4 [22%] female subjects), including 1 BAV (type A dissection) and 17 TAV patients. Type A AD occurred post-AVS in 3 TAV, non–Marfan syndrome patients: one received concomitant aorta plication at time of AVS (ascending aorta 45 mm) and the other 2 received AVS only (both had ascending aorta 47 mm). Twelve patients with type A AD were confirmed by emergent surgery, 3 confirmed by imaging studies, and 2 from death certificates.

The average dimensions of SOV, ascending aorta, maximal aorta size, and indexed maximal aorta size at the time of AD (12 patients had available data) were 53 ± 7 mm (range: 40-60 mm), 60 ± 10 mm (range: 45-80 mm), 60 ± 9 mm (range: 50-80 mm), and 35.5 ± 5.4 mm/m^2^, respectively. The annual growth rate of SOV and ascending aorta were 3.5 mm/year and 1.4 mm/year.

The overall cumulative AD incidence was 3.7% ± 1.0% at 10 years (incidence rate of 48.6 [95% CI: 29.7- 75.4] per 10,000 person-year); the corresponding cumulative AD incidence in TAV-AR was 4.8% ± 1.5% (Figure 1F). Baseline univariate predictors for AD (n = 18) are presented in Table 3. The dimension of SOV, indexed SOV, ascending aorta, and indexed ascending aorta were associated with AD risk but not age, BP, presence of hypertension, and Marfan syndrome; increased body mass index showed a trend of association. When we classified SOV with the reference value <45 mm, SOV 45 to 55 mm and SOV ≥55mm exhibited 5.7-fold and 5.5-fold risk of AD, respectively. When maximal aorta size was classified with the reference value <45 mm, similar results showed that patients with an aorta >45 mm had higher risks of AD in AR.

**Table 3:** Cox Proportional Hazards Model for Univariate Predictors of Aortic Dissection (N = 18)

	Hazard Ratio (95% CI)	*P* Value
Age, y	0.99 (0.97-1.02)	0.94
Systolic blood pressure, per mm Hg	1.00 (0.97-1.03)	0.75
Diastolic blood pressure, per mm Hg	0.97 (0.93-1.01)	0.16
Pulse pressure, mm Hg	1.01 (0.98-1.03)	0.24
Body mass index, kg/m^2^	1.10 (0.97-1.22)	0.10
Hypertension	1.35 (0.51-3.62)	0.53
Marfan syndrome	1.31 (0.17-9.87)	0.8
Sinus of Valsalva, mm	1.04 (1.00-1.08)	**0.026**
Sinus of Valsalva index, mm/m^2^	1.09 (1.00-1.16)	**0.029**
Sinus of Valsalva <45 mm (reference)		
45-55 mm	5.76 (1.99-16.6)	**0.001**
≥55 mm	5.53 (1.38-22.1)	**0.015**
Ascending aorta, mm	1.09 (1.04-1.13	**0.0001**
Ascending aorta index, mm/ m^2^	1.10 (1.03-1.17)	**0.004**
Maximal aorta size, mm	1.06 (1.03-1.09)	**0.0002**
Maximal aorta <45 mm (reference)		
45-55 mm	14.9 (3.1-72.1)	**0.0007**
≥55 mm	17.3 (3.73-80.2)	**0.0003**

**Bold** value indicates *P* value ≤0.05.

## Discussion

In this large contemporary Asian cohort, we compared differences between TAV and BAV patients with hemodynamically significant AR. Our major findings are: 1) compared with TAV-AR patients, BAV-AR patients were 2 decades younger, with similar absolute LV dimensions yet smaller BSA-normalized LV size, and were less symptomatic, resulting in lower AVS incidence; and 2) symptomatic TAV-AR patients had advanced cardiac remodeling, lower LVEF, higher pulse pressure, and tricuspid regurgitation pressure gradient. Conversely, except for higher systolic BP and lower LVEF, the associations between symptoms and cardiac remodeling were less prominent in BAV-AR. Other findings thus were: 1) although TAV-AR had a higher incidence of AVS, post-AVS LV recovery was good in both groups; 2) BAV-AR patients showed superior survival (mainly determined by age), and TAV-AR patients had excess risk of death compared with the general population, possibly caused by late referral; 3) TAV-AR patients had significantly larger BSA-normalized SOV and ascending aorta dimension, which led to a higher cumulative AD rate; and 4) maximal aorta size >45 mm was associated with higher AD risk.

### TAV-AR vs BAV-AR: differences between U.S. and taiwan cohorts

In our previous work from the United States, we found that, compared with TAV-AR patients, BAV-AR patients were less symptomatic and had good correlation between LV size and symptoms, a higher AVS incidence, more aortic valve repair, more concomitant aorta surgery, better overall survival caused by younger age, and survival similar to that expected.^3^ Comparison of major differences between Asians and Westerners is displayed in Supplemental Table 5 and the Central Illustration. This study showed universal findings of BAV and TAV differences from Asians and Westerners, including better overall survival in BAV (survival rate was comparable to BAV-AR in the U.S. cohort) because of age advantage^3^ and low AD risk in BAV,^12^, ^13^, ^14^ which were all reassuring. Also, post-AVS survival was good in both BAV and TAV and similar to that of the U.S. cohort. However, the interethnic differences from Western populations are evident: in addition to smaller BSA and more frequent BAV non–RL cusp fusion, TAV-AR patients in Taiwan had larger aorta size (particularly indexed value), reflected by high prevalence of aortic dilatation and less cusp prolapse as mechanisms for AR; eventually, this could potentially cause a higher AD rate in TAV-AR patients. Interestingly, in TAV-AR, there is a proportionate correlation between symptoms and chamber remodeling, different from the U.S. cohort,^2^ and cardiac remodeling was predictive of baseline symptoms. Of note, BP (pulse pressure in TAV and systolic BP in BAV) was predictive of baseline symptoms, highlighting the importance of attention to BP in patients with AR. However, the observation of lower AVS incidence in BAV-AR than TAV could be caused by a low rate of aortic valve repair in Taiwan because the need for prosthetic valve may cause hesitancy regarding AVS in young patients with BAV-AR. In the current study, we observed excess risk of death in TAV-AR compared with that in the general population, yet the TAV-AR survival-gap was larger herein compared with our prior work^3^ (Figure 1E). This may be explained (Supplemental Table 5) by our patients’ larger baseline indexed LV size (ie, at a relatively advanced stage in the AR disease course), larger aorta, and late surgical timing, partially because of smaller BSA in our patients, by nature, in which the LV remodeling was more likely overlooked.

**Central Illustration:**
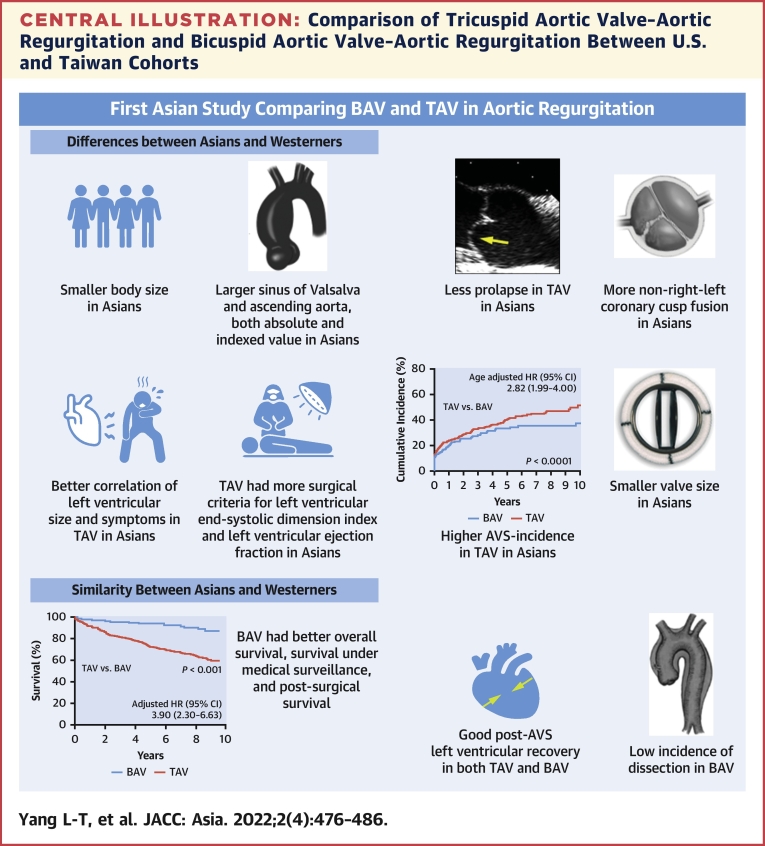
Comparison of Tricuspid Aortic Valve–Aortic Regurgitation and Bicuspid Aortic Valve–Aortic Regurgitation Between U.S. and Taiwan Cohorts Taiwanese patients had smaller body size, more non–right-left coronary fusion in bicuspid aortic valve (BAV), larger aorta size, and less cusp prolapse in tricuspid aortic valve (TAV). Baseline symptoms correlated better with chamber remodeling in TAV. Surgery-wise, aortic valve surgery (AVS) incidence was higher in TAV-AR than in BAV-AR, surgery was mainly driven by symptoms, repair rate was low, and a smaller prosthesis was used.^2^ Similarities between the Asian and U.S. cohort include comparable post-AVS survival, post-AVS left ventricular recovery, and low aortic dissection incidence in BAV patients.

### Risk of AD remained lower in BAV-AR than in TAV-AR in asia

For the first time, we report the cumulative incidence of AD in patients with chronic moderate to severe and severe AR. Most prior studies on the incidence of AD focus on patients with genetic aortopathy, including Marfan syndrome, BAV, and nonsyndromic thoracic aneurysms,^12^^,^^14^ but not patients with significant aortic valve disease. Although patients with BAV are frequently complicated by aortopathy, the notion that BAV patients had lower risk (≤1%) of AD than those with Marfan syndrome or other genetic-associated aortic aneurysms^12^^,^^14^^,^^15^ is well recognized. The current study also found a low rate of AD among BAV-AR, but the overall cumulative incidence of AD, driven by TAV-AR patients, was high in this study (3.7% ± 1.0% at 10 years; incidence rate of 48.6 [95% CI: 29.7-75.4] per 10,000 person-year). The incidence of AD in Taiwan was reported as 5.6 per 100,000 persons,^16^ which was conceivably lower than what we reported herein in patients with significant AR because our patients tended to have larger aorta size and more hypertension, placing them at higher risk of AD. Of note, Asian patients exhibited larger absolute and indexed aorta size, reflected by a higher rate of concomitant aorta surgery (46%) compared with our prior report in the United States (30%).^3^ Although we showed that baseline maximal aorta size (absolute or BSA-indexed) was linked to poor survival in TAV-AR, it is encouraging that AVS with concomitant aorta surgery seemed beneficial to survival (Supplemental Table 4, Supplemental Figure 3); this observation was also supported by a prior study.^17^ Most importantly, we showed that aorta size ≥45 mm (Table 3) was independently associated with AD risk, suggesting that surgical intervention could be considered at this point in patients with significant AR in both BAV and TAV, although prospective studies are required. Current guideline recommendations on a lower threshold (ie, 45 mm) for surgery on ascending aortic aneurysms included patients with Marfan syndrome plus risk factors,^18^ BAV patients undergoing AVS,^19^ or patients with small stature, rapid aorta progression, and AR.^18^ Our findings, which were from patients with relatively small BSA, are complementary to current guidelines on operative timing for aortic aneurysms.

### Clinical implications

From this large Asian cohort, important clinical implications are: first, clinicians can reassure BAV patients with AR about low AD risks during observation in the absence of other risk factors (ie, family history), similar life expectancy to general population, good post-AVS survival, and good LV recovery, which again emphasizes that age and not valve anatomy determined AR survival. Second, for patients with AR, regular TTE surveillance and clear reporting for AR progression and most importantly, aorta progression both before AVS and post-AVS is paramount because fatal AD may be prevented through early intervention once the aorta size was ≥45 mm. Third, attention should be paid to indexed LV size, not absolute LV size, in Asian patients with AR for timely surgical referral. In the face of culture-related surgery hesitancy, good post-AVS survival in both BAV-AR and TAV-AR patients should reassure the patient for prompt intervention.^20^ Lastly, the overall reduced survival of TAV-AR patients alerts clinicians from the communities in Asia to be more vigilant about significant AR and to refer patients earlier for timely intervention; educating the patients may improve their acceptance of AVS.

### Study limitations and strength

In this retrospective, tertiary referral center study, selection bias was possible. However, because of good accessibility to TTE and health care in Taiwan, our cohort reflected real-world conditions in patients with AR in Taiwan. The year of “baseline TTE” and related factors could play a role in outcomes; however, after adjusting it as a covariate (Supplemental Table 3), results were not changed. Confined to younger age (fewer deaths) and small sample size in BAV-AR, separate analysis for determinants of death in BAV was difficult. Images stored for proximal isovelocity surface area quantification for AR were not routinely obtained in our cohort, although low diastolic BP, similar pulse pressure as in our prior work,^21^ similar LV absolute size, larger indexed left ventricle, and post-surgical LV reverse remodeling all pointed to comparable AR severity, if not more, as in the U.S. cohort. Prevalence of concomitant aorta surgery was higher and indexed aorta size larger in Taiwan compared with the U.S. cohort; this phenomenon could be explained by late presentation in our cohort. We cannot rule out, however, the possibility of other causes for aorta disease as not every patient received genetic testing. Causes for symptoms were multifactorial (eg, comorbid conditions, AR severity). Also, the changes in LVEF and dimensions postsurgery, albeit significant, could be partially attributed to measurement variations. Finally, our national death records provided cause of death in all patients and allowed us to evaluate AD cumulative incidence although not every deceased patient underwent autopsy.

## Conclusions

Findings from this large contemporary Asian cohort comparing TAV-AR and BAV-AR are both reassuring and concerning. We showed that post-AVS survival in both groups and overall survival in BAV-AR were comparable to those of the U.S. cohort (Supplemental Table 5). However, compared with the U.S. cohort, TAV-AR patients had a larger survival gap; both BAV-AR and TAV-AR had larger absolute and indexed aorta size, larger indexed LV size, and lower AVS incidence. Also, the cumulative incidence of AD, which was reported for the first time in a significant AR cohort, was higher in TAV and seemed to be related to aorta size ≥45 mm. Therefore, in patients with AR (especially Asian subjects), more must be done to improve survival; use of indexed rather than absolute LV size to guide surgical referral merits re-emphasis, especially in TAV-AR. Aorta surgery could be considered, but more evidence is needed once the maximal aortic dimension is >45 mm to prevent fatal AD.

Perspectives **COMPETENCY IN MEDICAL KNOWLEDGE:** Differences between Asian TAV-AR and BAV-AR patients were not entirely the same as in the Western population. This cohort exhibited a larger baseline indexed left ventricle and aorta size for TAV-AR, suggesting that TAV-AR patients were at later stage of the AR natural course. Superior survival was noted in BAV-AR vs TAV-AR patients, who had a higher surgical incidence. Cumulative 10-year incidence of AD was higher in TAV-AR and was associated with baseline aorta size >45 mm.

**TRANSLATIONAL OUTLOOK:** Future research should explore whether a lower cutoff prompting concomitant aorta surgery in Asian patients with TAV-AR could improve outcomes by lowering the rate of AD.

## Funding Support and Author Disclosures

This study was supported by the Ministry of Science and Technology of Taiwan (MOST 110-2314-B-002-026) and the National Taiwan University Hospital (111-N0028). The authors have reported that they have no relationships relevant to the contents of this paper to disclose.

## References

[1] Michelena H.I., Prakash S.K., Della Corte A. (2014). Bicuspid aortic valve: identifying knowledge gaps and rising to the challenge from the International Bicuspid Aortic Valve Consortium (BAVCon). Circulation.

[2] Yang L.T., Michelena H.I., Maleszewski J.J., Schaff H.V., Pellikka P.A. (2019). Contemporary etiologies, mechanisms, and surgical approaches in pure native aortic regurgitation. Mayo Clin Proc.

[3] Yang L.T., Benfari G., Eleid M. (2021). Contemporary differences between bicuspid and tricuspid aortic valve in chronic aortic regurgitation. Heart.

[4] Coffey S., Roberts-Thomson R., Brown A. (2021). Global epidemiology of valvular heart disease. Nat Rev Cardiol.

[5] Kong W.K.F., Regeer M.V., Poh K.K. (2018). Inter-ethnic differences in valve morphology, valvular dysfunction, and aortopathy between Asian and European patients with bicuspid aortic valve. Eur Heart J.

[6] Hsing A.W., Ioannidis J.P. (2015). Nationwide population science: lessons from the Taiwan National Health Insurance Research Database. JAMA Intern Med.

[7] Lang R.M., Bierig M., Devereux R.B. (2005). Recommendations for chamber quantification: a report from the American Society of Echocardiography’s Guidelines and Standards Committee and the Chamber Quantification Writing Group, developed in conjunction with the European Association of Echocardiography, a branch of the European Society of Cardiology. J Am Soc Echocardiogr.

[8] Zoghbi W.A., Adams D., Bonow R.O. (2017). Recommendations for noninvasive evaluation of native valvular regurgitation: a report from the American Society of Echocardiography developed in collaboration with the Society for Cardiovascular Magnetic Resonance. J Am Soc Echocardiogr.

[9] Nishimura R.A., Otto C.M., Bonow R.O. (2014). 2014 AHA/ACC guideline for the management of patients with valvular heart disease: a report of the American College of Cardiology/American Heart Association Task Force on Practice Guidelines. J Am Coll Cardiol.

[10] Lee C.C., Lee M.G., Hsu T.C. (2018). A population-based cohort study on the drug-specific effect of statins on sepsis outcome. Chest.

[11] Robicsek F., Thubrikar M.J., Cook J.W., Fowler B. (2004). The congenitally bicuspid aortic valve: how does it function? Why does it fail?. Ann Thorac Surg.

[12] Weinsaft J.W., Devereux R.B., Preiss L.R. (2016). Aortic dissection in patients with genetically mediated aneurysms: incidence and predictors in the GenTAC Registry. J Am Coll Cardiol.

[13] Michelena H.I., Khanna A.D., Mahoney D. (2011). Incidence of aortic complications in patients with bicuspid aortic valves. JAMA.

[14] Sherrah A.G., Andvik S., van der Linde D. (2016). Nonsyndromic thoracic aortic aneurysm and dissection: outcomes with Marfan syndrome versus bicuspid aortic valve aneurysm. J Am Coll Cardiol.

[15] Yang L.T., Tribouilloy C., Masri A. (2020). Clinical presentation and outcomes of adults with bicuspid aortic valves: 2020 update. Prog Cardiovasc Dis.

[16] Yeh T.Y., Chen C.Y., Huang J.W., Chiu C.C., Lai W.T., Huang Y.B. (2015). Epidemiology and medication utilization pattern of aortic dissection in Taiwan: a population-based study. Medicine (Baltimore).

[17] Masri A., Kalahasti V., Svensson L.G. (2016). Aortic cross-sectional area/height ratio and outcomes in patients with a trileaflet aortic valve and a dilated aorta. Circulation.

[18] Erbel R., Aboyans V., Boileau C. (2014). 2014 ESC guidelines on the diagnosis and treatment of aortic diseases: document covering acute and chronic aortic diseases of the thoracic and abdominal aorta of the adult. The Task Force for the Diagnosis and Treatment of Aortic Diseases of the European Society of Cardiology (ESC). Eur Heart J.

[19] Hiratzka L.F., Creager M.A., Isselbacher E.M. (2016). Surgery for aortic dilatation in patients with bicuspid aortic valves: a statement of clarification from the American College of Cardiology/American Heart Association Task Force on Clinical Practice Guidelines. J Am Coll Cardiol.

[20] Chen S.W., Kuo C.F., Huang Y.T. (2020). Association of family history with incidence and outcomes of aortic dissection. J Am Coll Cardiol.

[21] Yang L.T., Enriquez-Sarano M., Scott C.G. (2020). Concomitant mitral regurgitation in patients with chronic aortic regurgitation. J Am Coll Cardiol.

