# Supplementary material for: Comparison Between Bicuspid and Tricuspid Aortic Regurgitation: Presentation, Survival, and Aorta Complications
Source: JACC Asia. 2022 Apr 2;2(4):476–86. doi: 10.1016/j.jacasi.2022.02.012 (PMC9627857; doi:10.1016/j.jacasi.2022.02.012)
Supplement: Supplemental Figures 1–3 and Tables 1–5 [file mmc1.docx]

**Supplemental Figure 1. Study flow**


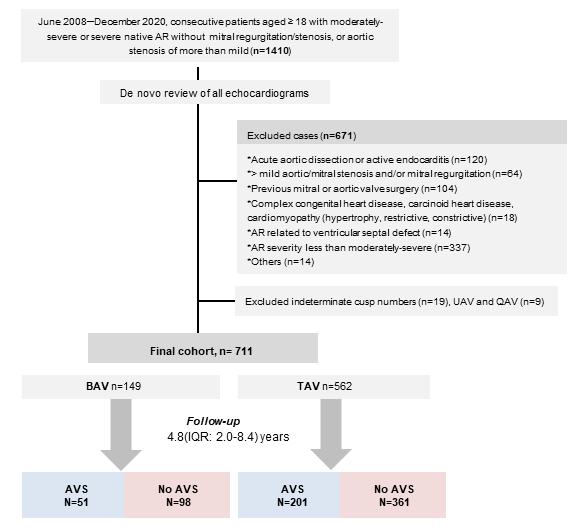


**Supplemental Table 1. Relationships Between Symptomatic Status and Findings From Echocardiogram**

|  | **Bicuspid aortic valve** | | | **Tricuspid aortic valve** | | |
| --- | --- | --- | --- | --- | --- | --- |
|  | Symptomatic  N=44 | Asymptomatic  N=100 | *P* | Symptomatic  N=285 | Asymptomatic  N=270 | *P* |
| Age, year | 55±17 | 45±15 | **0.001** | 71±14*** | 64±15 | **<0.0001** |
| SBP, mmHg | 143±17 | 133±18 | **0.003** | 137±19 | 133±19 | 0.05 |
| DBP, mmHg | 67±12 | 69±13 | 0.43 | 64±13 | 67±12 | **0.04** |
| Pulse pressure, mmHg | 74±17 | 66±19 | **0.019** | 72±21 | 67±18 | **0.001** |
| Charlson comorbidity index | 1.6±1.7 | 0.5±1.1 | **0.0001** | 2.0±2.0 | 1.1±1.6 | **<0.0001** |
| LVEF, % | 62±11 | 65±7 | **0.046** | 60±11 | 64±8 | **<0.0001** |
| LVEDD, mm | 62±8 | 61±7 | 0.19 | 60±8*** | 59±6* | 0.06 |
| LVEDDi, mm/m^2^ | 34.7±5.2 | 34.4±4.0 | 0.75 | 36.7±5 | 34.7±4.3 | **<0.0001** |
| LVESD, mm | 41±10 | 38±6 | 0.05 | 40±9 | 38±7 | **<0.0001** |
| LVESDi, mm/m^2^ | 22.8±5.8 | 21.6±3.4 | 0.17 | 24.6±5.6 | 22.1±4.1 | **<0.0001** |
| LVEDVi, ml/m^2^ | 111±41 | 109±33 | 0.85 | 102±39 | 92±32 | **0.002** |
| LVESVi, ml/m^2^ | 51±26 | 45±17 | 0.24 | 48.4±27 | 38±18 | **<0.0001** |
| LAVi, ml/m^2^ | 30±16 | 25±12 | 0.05 | 33±15 | 28±11 | **<0.0001** |
| TR PG, mmHg | 25±8 | 22±5 | 0.05 | 27±10 | 24±7 | **<0.0001** |
| E/e’ | 14±6 | 13±4 | 0.34 | 15±7 | 13±6 | **0.006** |
| ***Mechanisms*** |  |  |  |  |  |  |
| Any cusp prolapse | 14(34) | 62(67) | **0.0003** | 28(11) | 33(14) | 0.35 |
| Any cusp restriction | 15(37) | 9(8) | **0.0003** | 63(25) | 48(20) | 0.20 |
| Any dilatation | 36(82) | 82(86) | 0.49 | 224(85) | 228(90) | 0.08 |

Symptomatic status was available in 699 patients.

DBP, diastolic blood pressure; EF, ejection fraction LV; EDD(i), end-diastolic dimension (index); ESD(i), end-systolic dimension (index); EDV(i), end-diastolic volume (index); ESV(i), end-systolic volume (index); LV, left ventricular; RVSP, right ventricular systolic pressure; SBP, systolic blood pressure. Other abbreviations are as in Table 1.

*0.01≤ P<0.05, **0.001≤ P<0.01, ***P<0.001 in comparison between BAV and TAV in symptomatic and asymptomatic patients.

**Supplemental Table 2. Comparison Between Those With and Without Concomitant Aorta Surgery (n=249)***

|  | **Aorta surgery (+), n=114** | **Aorta surgery (-), n=135** | ***p*** |
| --- | --- | --- | --- |
| Age | 54±17 | 57±17 | 0.15 |
| Female | 21(18) | 21(15) | 0.49 |
| Hypertension | 63(56) | 69(51) | 0.38 |
| Hyperlipidemia | 21(18) | 20(15) | 0.42 |
| Diabetes mellitus | 8(7) | 11(8) | 0.75 |
| Atrial fibrillation | 8(7) | 6(4) | 0.35 |
| Connective tissue disease | 25(22) | 3(2) | **<0.0001** |
| Marfan syndrome | 21(19) | 0(0) | **<0.0001** |
| Coronary artery disease | 29(26) | 29(21) | 0.43 |
| Infective endocarditis | 3(3) | 10(7) | 0.07 |
| Charlson comorbidity index | 1.05±1.30 | 0.96±1.17 | 0.57 |
| Symptomatic | 52(46) | 83(60) | **0.03** |
| **Echo parameters** |  |  |  |
| Bicuspid aortic valve | 14(12) | 37(27) | **0.003** |
| LV ejection fraction, % | 59±11 | 62±9 | 0.05 |
| LV end-diastolic dimension, mm | 64±7 | 63±6 | 0.24 |
| LV end-diastolic dimension index, mm/m^2^ | 36±5 | 37±5 | 0.42 |
| LV end-systolic dimension, mm | 43±9 | 42±8 | 0.14 |
| LV end-systolic dimension index, mm/m^2^ | 24.3±5.2 | 24.3±5.2 | 0.91 |
| TR PG, mmHg | 24±7 | 24±8 | 0.56 |
| E/e’ | 14±7 | 15±6 | 0.82 |
| **Dimensions of aorta** |  |  |  |
| Annulus, mm(n=242) | 25.0±3.7 | 24.3±3.4 | 0.10 |
| Indexed Annulus(n=242) | 14.1±2.1 | 14.0±1.9 | 0.95 |
| Sinus of Valsalva, mm(n=240) | 51.3±11.1 | 39.7±5.1 | **<0.0001** |
| Indexed Sinus of Valsalva(n=240) | 28.7±5.8 | 22.9±4.1 | **<0.0001** |
| Mid-ascending aorta, mm(n=125) | 51.5±8.9 | 40.8±5.1 | **<0.0001** |
| Indexed Mid-ascending aorta(n=125) | 29.6±6.1 | 23.9±4.5 | **<0.0001** |

See Table 1 for abbreviations.

*Analysis excluded 3 patients: one received cardiac transplantation, 2 died intraoperatively.

**Supplemental Figure 2. LV Recovery After Surgery.** Post-surgical LV recovery was similar between BAV and TAV; while TAV patients had larger LVEF improvement due to lower baseline LVEFS. P value indicates comparison of %-change between BAV and TAV.**
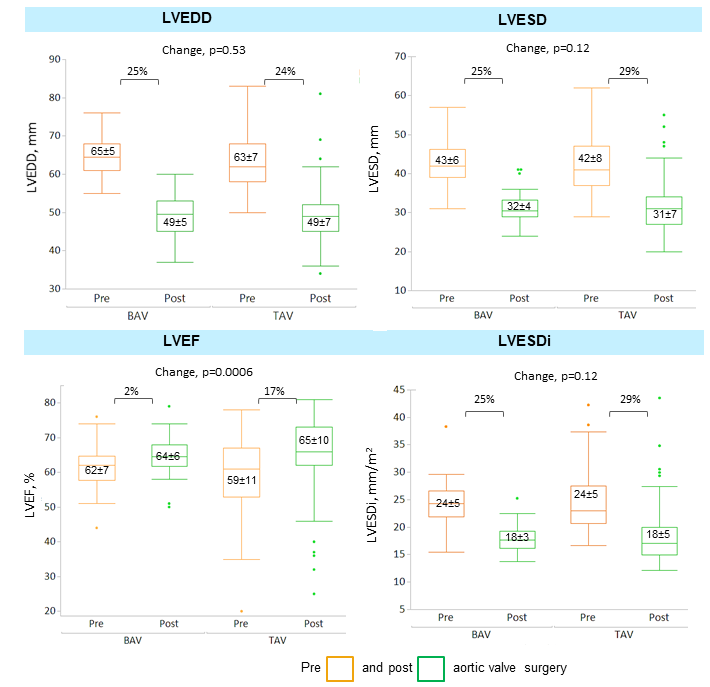
**

**Supplemental Figure 3. Survival According to Surgical Procedures.** Patients with aortic valve and aorta surgery (AVS+AoS) had better survival whereas those without surgery during total follow-up had the worst survival (A). As for post-surgical survival, those with AVS+AoS had less deaths as compared with those with AVS alone (B). When patients were further classified into BAV or TAV, there were inter-group survival differences, mostly driven by TAV patients (6 deaths in those having AVS+AoS; 20 deaths in those having AVS alone) (C). There were only 2 post-surgical deaths in BAV-AR, therefore, the impact of AVS+AoS and AVS alone on post-surgical survival in BAV-AR was uncertain due to limited statistical power (C).

**
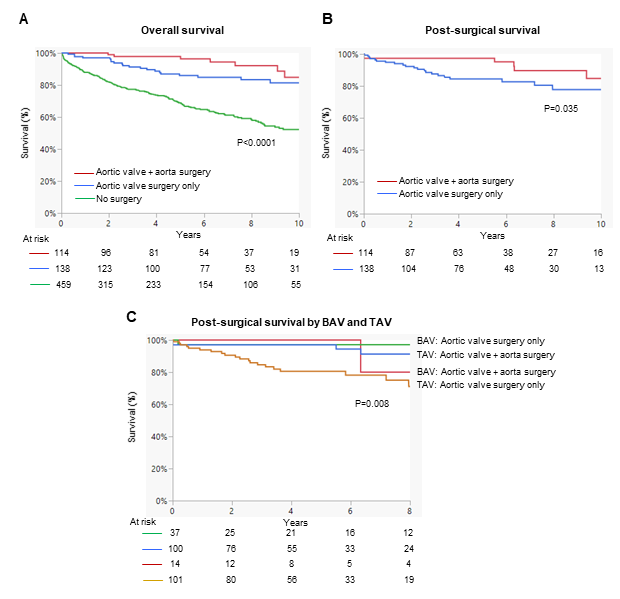
**

**Supplemental Table 3. Cox Proportional Hazard Models for Determinants of Overall Survival**

|  | **HR (95% CI)** | **P** |
| --- | --- | --- |
| **Model-1** |  |  |
| Age, per year | 1.06(1.04-1.07) | <0.001 |
| Male | 0.64(0.46-0.89) | 0.008 |
| Charlson Comorbidity Index | 1.23(1.14-1.33) | <0.001 |
| LV ejection fraction, per 10% decrease | 0.81(0.71-0.94) | 0.005 |
| NYHA I (Reference) |  |  |
| II | 1.28(0.90-1.81) | 0.15 |
| III-IV | 2.04(1.26-3.32) | 0.003 |
| BAV vs TAV | 0.95(0.53-1.69) | 0.87 |
| Time dependent AV surgery | 0.58(0.37-0.92) | 0.02 |
| **Model-2** |  |  |
| Age, per year | 1.06(1.04-1.07) | <0.001 |
| Male | 0.62(0.45-0.87) | 0.005 |
| Charlson Comorbidity Index | 1.25(1.16-1.35) | <0.001 |
| LV ejection fraction, per 10% decrease | 0.82(0.72-0.95) | 0.008 |
| NYHA I (Reference) |  |  |
| II | 1.30(0.92-1.84) | 0.13 |
| III-IV | 2.17(1.34-3.54) | 0.001 |
| BAV vs TAV | 0.94(0.53-1.66) | 0.83 |
| Time dependent AV surgery | 0.61(0.39-0.97) | 0.03 |
| Year of baseline TTE(continuous variable) | 0.94(0.89-0.99) | 0.02 |

NYHA, New York Heart Association functional class; TTE, transthoracic echocardiogram. See Table 1 for other abbreviations.

**Supplemental Table 4. Cox Proportional Hazard Models for Determinants of Survival.**

|  | **Hazard ratio(95% CI)** | ***P*** |
| --- | --- | --- |
| 1. **Determinants for survival under medical surveillance in TAV (170 deaths)** | | |
| **Model-1** |  |  |
| Age, years | 1.06(1.05-1.09) | **<.0001** |
| Charlson comorbidity index | 1.24(1.15-1.34) | **<.0001** |
| Female | 1.77(1.24-2.52) | **.002** |
| Left ventricular ejection fraction, % | 0.97(0.96-0.99) | **.010** |
| **Maximal aorta size, mm** | 1.02(1.00-1.05) | **.042** |
| **Model-2** |  |  |
| Age, years | 1.06(1.04-1.08) | **<.0001** |
| Charlson comorbidity index | 1.25(1.16-1.35) | **<.0001** |
| Female | 1.42(0.99-2.05) | .059 |
| Left ventricular ejection fraction, % | 0.98(0.96-0.99) | **.026** |
| **Indexed maximal aorta size, mm/m^2^** | 1.05(1.02-1.09) | **.003** |
| 1. **Determinants for postsurgical survival in the total cohort (28 deaths)** | | |
| Age, years | 1.05(1.02-1.09) | **.0005** |
| Charlson comorbidity index | 1.45(1.15-1.77) | **.002** |
| AVS + aorta surgery vs. AVS alone | 0.45(0.19-1.08) | .07 |

AVS, aortic valve surgery

**Supplemental Table 5. Comparison Between Taiwan and US Cohort Regarding BAV-AR Versus TAV-AR**

|  | **Taiwan** | | **US** | |
| --- | --- | --- | --- | --- |
|  | **BAV, n=149** | **TAV, n=562** | **BAV, n=296** | **TAV, n=502** |
| **Baseline demography** |  |  |  |  |
| Age, year | 48±16 | 68±15 | 46±14 | 67±14 |
| Female | 14(9) | 148(26) | 19(6) | 122(24) |
| Body surface area, *m^2^* | 1.78±0.19 | 1.68±0.20 | 2.07±0.21 | 1.97±0.24 |
| NYHA I* | 100(69) | 270(49) | 200(69) | 256(54) |
| II | 40(29) | 213(38) | 75(26) | 141(30) |
| III+IV | 4(2) | 72(13) | 16(5) | 79(16) |
| **Echo parameters** |  |  |  |  |
| BAV fusion, RL fusion | 72% |  | 82% |  |
| RN | 23% |  | 17% |  |
| LN | 5% |  | 1% |  |
| LVEDDi, mm/m^2^ | 34.4±4.4 | 35.7±4.8 | 30.2±3.7 | 30.2±4.1 |
| LVESDi, mm/m^2^ | 21.9±4.3 | 23.3±5.0 | 20.0±3.4 | 20.3±3.9 |
| LVESDi>25 mm/m^2^ | 26(17) | 154(27) | 21(7) | 63(13) |
| LVEDVi, ml/m^2^(n=700) | 107±36 | 98±37 | 112±30 | 98±29 |
| LVESVi, ml/m^2^(n=698) | 46±21 | 43±25 | 48±19 | 42±18 |
| **Aorta dimensions** |  |  |  |  |
| Annulus, mm | 25.9±3.7 | 22.9±3.0 | 28.0±2.9 | 24.7±2.8 |
| Indexed Annulus | 14.7±2.3 | 13.7±1.9 | 13.6±1.8 | 12.6±1.4 |
| Sinus of Valsalva, mm | 40.3±5.6 | 42.0±8.9 | 40.5±5.3 | 40.1±6.2 |
| Indexed Sinus of Valsalva | 22.8±3.7 | 25.1±5.2 | 19.6±2.9 | 20.5±3.2 |
| Mid-ascending aorta, mm | 42.6±7.7 | 44.3±8.1 | 40.1±6.7 | 41.2±7.9 |
| Indexed Mid-ascending aorta | 23.9±4.8 | 26.5±5.6 | 19.5±3.6 | 21.0±4.8 |
| **Surgical data(n=51/198/167/236)** | | | | |
| Surgery for symptoms | 27(53) | 166(83) | 95(57) | 175(74) |
| Surgery for LVESD(i) >50mm(25mm/m^2^) | 7(13) | 13(6.5) | 19(12) | 38(17) |
| Aortic valve repair | 1 (-) | 7(-) | 48(29) | 48(20) |
| Concomitant aorta surgery | 14(27) | 100(50) | 64(38) | 59(25) |
| Concomitant coronary artery bypass grafting | 4(8) | 25(12) | 4(2) | 52(22) |
| Valve size^†^ |  |  |  |  |
| <23 mm | 2(5) | 18(11) | 5(4) | 25(13) |
| 23,24 mm | 15(36) | 76(44) | 8(7) | 20(11) |
| 25 mm | 24(57) | 71(41) | 36(30) | 66(35) |
| >25 mm | 1(2) | 7(4) | 70(59) | 77(41) |
| **Post-AVS LV recovery** |  |  |  |  |
| Change of LVEDD after AVS | 25% | 24% | 16% | 14% |
| Change of LVESDi after AVS | 25% | 29% | 16% | 15% |
| **Surgical incidence and survival at 8-year^‡^** |  |  |  |  |
| Incidence of AVS | 36±4% | 47±3% | 60±3% | 53±3% |
| Survival of total follow-up  (%, HR, 95% CI) | 90±3%  0.25(0.14-0.43)^§^ | 63±3%  reference | 93±7%  0.19(0.11-0.30)^§^ | 71±2%  reference |
| Survival under medical surveillance  (%, HR, 95% CI) | 89±3%  0.22(0.12-0.39)^§^ | 54±3%  reference | 89±4%  0.20(0.10-0.39)^§^ | 61±4%  reference |
| Post-AVS survival  (%, HR, 95% CI) | 93±5%  0.30(0.07-1.26)^¶^ | 81±4%  reference | 94±2%  0.20(0.09-0.44)^§^ | 77±3%  reference |
| Survival compared to general population  (HR and P value) | 1.8, P=0.08 | 3.1, P<0.0001 | 1.2, P=0.49 | 1.2, P=0.05 |

AVS, aortic valve surgery; CI, confidence interval; LVEDD, LV end-diastolic dimension; LVESD, LV end-systolic dimension; NYHA, New York Heart Association functional class; HR, hazard ratio.

Other abbreviations are as in Table 1 and Supplemental Table 1.

*Patients with undetermined NYHA status were excluded.

^†^Of 241 patients having AV replacement, valve size were unknown in 27 patients who received AVS outside.

**^‡^**For ease of comparison, data at 8-year was shown.

^§^P<0.0001, ^¶^P>0.05
